# Decellularized Tissue for Muscle Regeneration

**DOI:** 10.3390/ijms19082392

**Published:** 2018-08-14

**Authors:** Anna Urciuolo, Paolo De Coppi

**Affiliations:** 1 Department of Industrial Engineering, University of Padova, 35122 Padova, Italy; anna.urciuolo@unipd.it; 2 Great Ormond Street Institute of Child Health, University College of London, London WC1N 1EH, UK

**Keywords:** skeletal muscle engineering, tissue engineering, volumetric muscle loss, decellularized tissue, decellularized muscle, acellular tissue, acellular muscle, skeletal muscle regeneration

## Abstract

Several acquired or congenital pathological conditions can affect skeletal muscle leading to volumetric muscle loss (VML), i.e., an irreversible loss of muscle mass and function. Decellularized tissues are natural scaffolds derived from tissues or organs, in which the cellular and nuclear contents are eliminated, but the tridimensional (3D) structure and composition of the extracellular matrix (ECM) are preserved. Such scaffolds retain biological activity, are biocompatible and do not show immune rejection upon allogeneic or xenogeneic transplantation. An increase number of reports suggest that decellularized tissues/organs are promising candidates for clinical application in patients affected by VML. Here we explore the different strategies used to generate decellularized matrix and their therapeutic outcome when applied to treat VML conditions, both in patients and in animal models. The wide variety of VML models, source of tissue and methods of decellularization have led to discrepant results. Our review study evaluates the biological and clinical significance of reported studies, with the final aim to clarify the main aspects that should be taken into consideration for the future application of decellularized tissues in the treatment of VML conditions.

## 1. Introduction

The crucial role of the ECM environment in the stem cell niche, and in the regulation of stem cell identity and differentiation, organogenesis and tissue homeostasis has been a topic of extensive and intriguing study [1,2]. In the field of regenerative medicine this has allowed for the development of an increasing number of tissue engineering strategies, in which scaffold materials are used to mimic in vivo the biological microenvironment of the ECM, providing the components needed to drive cells toward the regeneration of the tissue of interest. Despite the incredible improvements that have been made in 3D bioprinting technology, the bona-fide reproduction of a scaffold capable to accurately mimic complex tissues, such as skeletal muscle, remains a matter that cannot be technically solved [2]. The 3D interactions existing among different components of the ECM is far for being a simple overlay of proteins organized in a layer-by-layer fashion. Indeed, ECM components not only interact with each other in specific fashions, but each single component, and also defined isoforms of a same component, are tissue-specific and even site-specific inside a defined tissue [2,3]. Such complexity, which likely has a biological meaning for cells, can be preserved in scaffolds only by taking advance of the native tissue themselves, that is achieved by decellularizing tissues or whole organs. Upon the removal of nuclear content and cellular elements, decellularized or acellular scaffolds still retain the architecture and complexity of the native tissues, including vasculature and biofactors present in the ECM. These characteristics make decellularized matrices the ideal bio-scaffold necessary to guide host or donor cells toward the regeneration of new and functional tissues. Several studies have already demonstrated the possibility of successfully obtaining acellular scaffolds from many organs, such as heart, kidney, pancreas, lung, liver, esophagus and intestine. Importantly, some of these decellularization protocols have been adopted to decellularize simple hollow organs such as the trachea, which have then been successfully transplanted in patient after autologous cell seeding, i.e., trachea [4,5,6]. Importantly, the trachea transplant has been achieved without immunosuppression, a great advantage over conventional transplantation because it avoids potential risks for patients, including frequent infections and cancer. Acellular tissues are biocompatible and the absence of rejection after allogeneic or xenogeneic transplantation make them the ideal scaffold for translational medicine applications and organ replacement [7,8]. 

Even though skeletal muscle has a remarkable capacity to undergo regeneration, several pathological conditions can lead to extensive and irreversible muscle loss: i.e., congenital defects, traumatic injuries, surgical ablations, and neuromuscular diseases [2]. Failure of normal regeneration results in VML, with loss of muscle function, often associated with scar tissue formation and adipose tissue substitution. Current treatments for such conditions have limited success [7], leading to considerable social and economic burden. Therefore, there is a great need for new regenerative medicine strategies aimed at treating VML conditions. Skeletal muscle is a complex tissue in which myofiber 3D organization and function is intimately linked to other tissue components, such as motor neurons, vasculature, myogenic stem cells (including satellite cells, SCs), interstitial cells, and ECM [9,10,11]. A tubular network of ECM is intimately connected to all the different cellular components. However, the ECM does not just provide mechanical support for bearing force transmission, but also a dynamic signaling environment that is crucial for muscle development, homeostasis, and regeneration [12,13]. SCs are mitotically quiescent muscle stem cells necessary for muscle regeneration and located between the basal lamina and myofibers [11]. Defined composition and mechanical properties of the ECM in SC niche is required to allow SC self-renewal and efficient muscle regeneration in vivo [11,14,15,16,17,18]. 

## 2. Acellular Tissues and Biomaterials for VML Treatment: Types and Methods

The ideal biomaterial for VML repair would need to fill the volumetric loss and sustain SC activity, while guaranteeing access to both vascular and neural cells for a correct revascularization and reinnervation of the tissue [7,8,19]. Based on the above points, it is not surprising that acellular scaffolds derived form a range of different tissues have already been tested in animal models [20,21,22,23,24,25,26,27,28,29,30,31,32,33,34] and in small cohorts of patients affected by VML [35,36]. 

Acellular tissues are mainly generated by using physical, enzymatic, and/or chemical mechanisms [9,37,38,39]. Based on its simplicity, the most commonly used method to obtain acellular tissue is immersion and agitation of the sample, in presence of decellularizing agents. However, this approach does not allow homogeneous decellularization of large samples and thick tissues, such as skeletal muscle. To overcome such limitation, perfusion methods have been developed. Indeed, by using the native vascular tree of the tissue/organ, decellularizing reagents can be homogeneously distributed across the tissue, allowing better access and deep tissue exposure, ultimately improving the removal of cellular components from large tissues [40,41,42]. Jank and colleagues reported the ability to decellularize rat and primate forearms by perfusion of detergents. The preservation of the composite architecture allowed the repopulation of muscle and vasculature of the construct with cells of appropriate phenotypes in vitro, and also supported blood perfusion following in vivo transplantation [43]. Moreover, a recent report by Gerli and colleagues also successfully generated a large-scale, acellular composite tissue scaffold from a full cadaveric human upper extremity using a perfusion method of decellularization [44]. This construct retained its morphological architecture and perfusable vascular conduits with the preservation of the native ECM components. Such biocompatible constructs could have significant advantages over the currently implanted matrices, in terms of nutrient distribution, size-scalability, and immunological response [44]. These studies demonstrate the possibility of developing more complex and reproducible decellularized organs, which hold higher regenerative potential and translational efficacy for the treatment of VML conditions. 

Small intestine submucosa matrix (SIS), urinary bladder matrix (UBM), and skeletal muscle decellularized scaffolds have been the most commonly used materials to repair VML defects. SIS and UBM scaffolds are prepared with standardized protocols, and are commercially available, clinically approved, and have also been used in patients [30]. Conversely, skeletal muscle scaffolds have only been used in animal models, and their preparation has been shown to be a more complex procedure, mainly due to tissue complexity and thickness. The main protocols applied to generate decellularized scaffolds from skeletal muscle are essentially of three types: enzymatic-not detergent [9,24,29], detergent [31,32,33,34], and detergent-enzymatic [33,45,46] treatments. In the literature such scaffolds are defined as decellularized; however, some of them would be better defined as “anucleated” rather than “acellular” scaffolds. Indeed, together with the elimination of the nuclear content and the maintenance of the ECM, such treatments can partially preserve cytoplasmic components of the original tissue, in particular those belonging to myofibers [24,32,33,39,45,46].

As mentioned above, different sources of tissues and methods of decellularization have been used to generate acellular scaffolds for the treatment of VML, complicating the understanding of which scaffold is most ideal. The biological properties of the scaffolds depend on the agent, or combined-agents, used for inducing decellularization, as well as on the method applied (i.e., immersion vs. perfusion) and on the characteristics of the primary tissue/organ (density, cellularity, dominant component in tissue, and thickness) [37]. Another important factor to consider is whether using a tissue-matched decellularized scaffold can have positive effects on regeneration. SIS and UBM are scaffolds that can fill the volumetric loss of tissue but do not have any muscular specific components. Based on the complexity of the biological meaning of ECM—discussed above— it is reasonable to speculate that matched acellular tissues should be better at instructing host and/or seeded-donor cells toward the regeneration of the tissue of interest. 

While this is not the focus of the present work, it is important to recognize that major work in the field of skeletal muscle tissue engineering has been undertaken using polymers. Polymers have some advantages over decellularized tissue, e.g., their manufacture is more reliable and consistent, allowing a precise design of their geometry as well as mechanical and structural properties. As with natural scaffolds, they can be loaded with bioactive molecules. Scaffolds used for skeletal muscle regeneration include (i) synthetic polymers such as poly(ethylene glycolic) (PEG), polyglycolic acid (PGA), poly(lactic-co-glycolic acid) (PLGA), poly(l-lactic acid) (PLLA), their copolymers (PLLA/PLGA), and polycaprolactone (PCL); (ii) natural polymers such as alginate, collagen, fibrin, or hyaluronic acid, or (iii) a combination of the two [47,48,49,50,51,52,53,54,55,56,57,58,59,60]. Rat myoblasts seeded onto PGA meshes and implanted into the omentum of syngeneic animals generated vascularized constructs in which myoblasts were found to be organized between strands of degrading polymer mesh [48,49]. In comparison to synthetic materials, natural polymers may have the advantage of closely resembling the original ECM, or ECM function of a tissue, and will thus facilitate an efficient regeneration. For example, fibrin itself can efficiently promote regeneration in skeletal muscle. Fibrin microthreads seeded with adult human cells improved regeneration of a large defect in the tibialis anterior muscle in murine model of VML, with new muscle tissue formation and presence of Pax7-positive cells [55]. Efficient muscle regeneration using fibrin was also previously demonstrated using a 3D fibrin matrix seeded with expanded primary rat syngeneic myoblasts [56]. Another chief component of the ECM, hyaluronan, has been shown to promote muscle regeneration when seeded with muscle progenitor cells in a murine model of VML. Notably, SCs embedded in photo-cross-linkable hyaluronan also promoted functional skeletal muscle recovery, with the formation of both neural and vascular networks and the reconstitution of a functional SC niche [57]. Growth factors can also be efficiently associated to synthetic or natural polymers. Mouse or human mesoangioblasts engineered to express placental-derived growth factor and encapsulated inside PEG-fibrinogen hydrogels were able to efficiently replace an ablated tibialis anterior [61]. In another study, an injectable, degradable alginate gel loaded with vascular endothelial growth factor (VEGF) and insulin-like growth factor 1 (IGF1) led to parallel angiogenesis, reinnervation, and myogenesis upon ischemic damage in skeletal muscle, with SCs activation, proliferation, and simultaneous protection from inflammation and apoptosis [51]. VEGF can also be delivered by genetically engineering grafted cells. Rat myoblasts transfected with a plasmid encoding VEGF and suspended in collagen type I showed increased generation of tissue mass after subcutaneous injection into nude mice compared with nonfunctional VEGF-transfected cells [52]. Besides using factors promoting angiogenesis, factors that induce cell activation and migration, such as hepatocyte growth factor (HGF) and fibroblast growth factor (FGF), can also promote scaffold grafting and myogenesis [53]. The role of growth factors and natural polymers was further investigated by a recent paper in which VEGF, IGF-1, FGF, HGF, and other factors were released locally from alginate microbeads. Such constructs induced differentiation of urine-derived stem cells into a myogenic lineage, enhanced revascularization and innervation of the implants and stimulated in vivo resident cell growth [54]. 

## 3. VML Models for Testing Acellular Tissues

The translational potential of decellularized tissues has mainly been evaluated in vivo using surgical animal models of VML. Therefore, the use of such models mainly aim to test the ability of decellularized tissues to promote (i) cell homing; (ii) regeneration of myofibers and motor neuron axons; and (iii) angiogenesis. These are all essential steps necessary to have a functional skeletal muscle in the site of transplantation. Moreover, acellular tissues have been used as natural devices in which host cells can drive muscle regeneration, as well as used as scaffolding material to deliver donor cells with the aim to improve cell therapy strategies. In order to evaluate the studies that have been reported so far, here we compare the results obtained among different acellular scaffolds and strategies—i.e., with or without donor cells, used for the treatment of VML in comparable animal models (Table 1).

Mice are commonly used for studying muscle regeneration and testing the regenerative potential of acellular scaffolds in VML. Sicari and colleagues studied the ability of porcine SIS to promote muscle regeneration after xenotransplantation in a VML model in which the quadriceps muscle was ablated. Although no details were reported in terms of functional activity of the implant, the authors concluded that the scaffold within the defect was associated with constructive tissue remodeling, including the formation of site-appropriate skeletal muscle tissue [26]. The same model was used in another study to test the regenerative potential of porcine UBM. In this instance, Fisher and colleagues showed that the scaffolds promoted the formation of functional skeletal muscle cells, with perivascular stem cell mobilization and their accumulation within the site of injury [35]. Yet another study reported the ability of sodium dodecil sulfate (SDS)-derived acellular skeletal muscle to promote the formation of islands of myofibers after implantation in a tibialis anterior VML model [21]. Recently, few studies strongly supported the idea that decellularized muscles can offer a favorable environment to donor or host cells that promotes functional muscle regeneration [31,33,34]. We recently demonstrated that decellularized skeletal muscles derived with three different perfusion methods per se were able to generate functional artificial muscles in a xenogeneic immune-competent model of VML, in which the EDL muscle was surgically resected. In particular, decellularized tissues promoted migration and differentiation of the host myogenic cells, as well as SC homing, the formation of nervous fibers, neuromuscular junction and vascular networks [33]. Quarta and colleagues showed that decellularized muscle seeded with adult muscle stem cells and muscular resident cells were able to generate functional muscle tissue in a VML model. More in detail, SDS-based acellular muscles were used to deliver and promote the maintenance of human muscular cells in an immunocompromised murine model of VML in which the tibialis anterior muscle was ablated. Both innervation and in vivo force production were enhanced when implantation of bioconstructs was followed by an exercise regimen [31]. Another study showed the ability of decellularized skeletal muscle to support functional muscle regeneration by host cells, with less fibrosis and more de novo neuromuscular receptors than either autograft or collagen implants in a rat model of gastrocnemius VML [32].

The rat represents the most commonly used animal model for assessing muscle regeneration upon treatment of VML with acellular tissues. More than 10 years ago, we used a full-thickness defect of the abdominal wall to evaluate the syngeneic regenerative potential of cell-matrix constructs composed of SC–derived myoblasts seeded on muscle acellular matrices. Acellular muscles were obtained by a detergent-enzymatic method, and showed the preservation of both FGF and transforming growth factor-beta. The implanted construct promoted the formation of skeletal muscle and allowed the survival of donor cells nine months after surgery. Interestingly, a vesicular acetylcholine transporter was present on the surface of muscle fibers identified in the implant, suggesting the possible integration of the nervous system [22]. A similar approach was performed later in a xenogeneic model. Fishman and colleagues used a VML model of tibialis anterior for the implantation of a construct composed by rat skeletal muscle scaffolds and murine myoblasts. The decellularized muscle was prepared using an enzymatic protocol performed under agitation. Unfortunately, the aim of the study was focused on the immunomodulatory activity of the scaffold and muscle regeneration at the site of implantation was poorly characterized. However, the authors demonstrated that donor cells displayed better survival when delivered thought the scaffold, adding relevant information regarding the use of acellular matrix as scaffolding material for cell therapy approaches [24]. More recently, another study tested the possible application of UBM as scaffolding material for syngeneic cell delivery. Goldman and colleagues combined UBM with minced muscle grafts, promoting de novo muscle fiber regeneration and neuromuscular strength recovery in a VML model in which the tibialis anterior muscle was ablated. However, this functional improvement was associated with a concomitant reduction in graft-mediated regeneration, with coincident fibrous matrix deposition and interspersed islands of nascent muscle fibers. This effect was linked to the inability of UBM to create a favorable environment for efficiently promoting muscle regeneration [25]. Such results indicate that acellular scaffolds can ameliorate the survival of delivered cells. However, we can speculate that acellular muscles are better than UBM scaffolds when it comes to support muscle regeneration and donor cell maintenance. 

Studies aimed at using acellular scaffolds with no implementation of cellular therapy have also been reported in rat VML models. Merritt and colleagues derived skeletal muscle decellularized tissues from rat by using an SDS-based immersion protocol. The scaffolds were implanted in a model in which a portion of the lateral gastrocnemius had been removed. After 42 days, growth of blood vessels and myofibers into the ECM was apparent, but no restoration of force occurred [23]. In another study, the histomorphologic characteristics and the physiologic activity of the implants were evaluated in an abdominal wall VML model upon implanting either porcine SIS, carbodiimide-cross-linked porcine SIS, autologous tissue, or polypropylene mesh. The best histomorphologic results were obtained with SIS scaffold, which showed islands and sheets of skeletal muscle. On the other hand, functional recovery was similar between SIS- and autologous tissue-implants. Conversely, implants of carbodiimide-cross-linked SIS and polypropylene mesh were characterized by a chronic inflammatory response and produced little or no measurable tetanic force [29]. Despite the range of final responses, these studies also supported the idea that acellular tissues can per se promote muscle regeneration in VML models.

Muscular or nonmuscular decellularized tissues implanted in the same VML model have been used to directly compare their ability to promote muscle regeneration. Wolf and colleagues used a partial thickness abdominal wall defect model in which they xeno-transplanted muscle-derived scaffold or SIS-derived matrix. In this study, the muscular tissue was derived from dogs and decellularized muscles were obtained with an agitation method and an enzymatic and chemical decellularization process. Even though acellular muscles were shown to have preserved growth factors, glycosaminoglycans, and basement membrane structural proteins, which differed from those present in SIS, the remodeling outcome was comparable between the two implanted scaffolds [20]. On the other hand, a different study reported that upon xenotransplantation decellularized muscles allowed better neovascularization, myogenesis, and functional recellularization than that obtained with porcine SIS implants. Interestingly, the muscular scaffolds used in this particular study, prepared from porcine rectus abdominis, were obtained with a perfusion method and retained the intricate 3D microarchitecture and vasculature networks of the native tissue, many of the bioactive ECM components and the mechanical properties [28]. Altogether these results strongly support the idea that the protocol used to decellularize skeletal muscle is fundamental in determining if and to what extent the obtained scaffold is capable to improve the regenerative response of the hosting tissue.

It is important to underline that the large volume of tissue that needs to be regenerated in patients affected by VML can be a major limiting step for acellular tissue application in clinic. To address this point, some studies attempted to apply decellularized matrix to larger animal models. Turner and colleagues have used a canine VML model, in which the distal third of the vastus lateralis and a portion of the vastus medialis muscles were resected. This defect was replaced with a scaffold composed of SIS. Even though the initial remodeling process followed a pattern similar to that reported in other studies, in the long term the implanted scaffolds showed dense collagenous tissue formation and islands of skeletal muscle with no successful restoration of tissue functionality [27]. In a more recent study, similar results were obtained in a porcine VML model in which peroneous tertius muscle was ablated. By comparing the outcome of implanted SIS, UBM, and hyaluronic acid hydrogel, Greising and colleagues showed that in all three cases ECM implantation could not orchestrate a skeletal muscle regeneration capable to lead to a physiological improvement. Instead, a significant deposition of fibrotic tissue was observed within the defect region three months post-injury [30]. Still, SIS and UBM have been shown to allow functional improvement in patients affected by VML. In particular, Fisher and colleagues showed that functional improvement was observed in three out of the five patients after implantation of SIS scaffolds [35]. More recently, a 13-patient cohort study was performed, using commercially available ECMs—BioDesign (SIS), Matristem (UBM), and Xenatrix (dermis-ECM)—to repair VML defects. The authors reported that in all cases the acellular scaffold facilitated functional tissue remodeling, thanks to the recruitment of myogenic progenitor cells and improved innervation [32]. These clinical results are in contrast with those obtained from studies performed in large animal models [27,30]. More studies will be needed to determine if the reason for such discrepancies is related to species-specific biologic responses to decellularized scaffold implantation. Besides, it is also important to emphasis that so far muscular acellular scaffolds have not been tested in large animal models or patients.

## 4. Conclusions and Perspectives

Studies performed on murine and rat VML models have conclusively demonstrated the ability of acellular scaffolds to promote myogenesis both as stand-alone devices and when associated with cell therapy strategies. Interestingly, by comparing results obtained between this two animal models one could speculate that acellular scaffolds derived from skeletal muscle are to be the best candidate to promote skeletal muscle regeneration in vivo, when compared to SIS and UBM scaffolds. This conclusion seems to be valid not only when scaffolds are used as devices, but also when they are associated with cell therapy. The possibility of using decellularized scaffolds as devices represent an important aspect for their translational application, as it eliminates the limiting steps specifically related to cell therapy. Perfusion methods of decellularization appear to better preserve instructive cues necessary to promote functional muscle regeneration by host cells. The recently published method of composite tissue decellularization, strongly suggest the feasibility of applying such muscular scaffolds in large animals and patients [43,44].

Overall, we can therefore conclude that despite incredible improvements in the design and development of synthetic or natural materials, decellularized tissues possess the irreplaceable advantage of preserving the biological signals that mimics the normal ECM of an in vivo tissue. Hence, we strongly believe that for the next foreseeable future application of decellularized tissues will represent a valid and powerful strategy to develop new therapeutic approaches for the cure of VML conditions.

## Figures and Tables

**Table 1:** Decellularized tissues used in vivo. The table reports the studies in which decellularized tissues have been applied in vivo. The type of decellularized tissues, the method of decellularization, the type of the eventual seeded cells, the species subject to implantation, and the in vivo outcome have been reported for each reference. –, not performed.

Decellularized Tissue	Method of Decellularization	Seeded Cells	Mplanted Species	In Vivo Outcome	Ref.
Porcine small intestine submucosa (SIS); Canine skeletal muscle	Immersion	–	Rat	Remodeling and partial skeletal muscle regeneration. Comparable results between SIS and skeletal muscle scaffolds	[21]
Murine skeletal muscle	Immersion	–	Mouse	Remodeling and partial skeletal muscle regeneration	[22]
Rat skeletal muscle	Immersion	Rat SC–derived myoblasts	Rat	Partial skeletal muscle regeneration	[23]
Rat skeletal muscle	Immersion	–	Rat	Partial skeletal muscle regeneration. No force restoration	[24]
Rat skeletal muscle	Immersion	Murine myoblasts	Rat	Improvement of donor cells survival	[25]
Porcine urinary bladder matrix (UBM)	Immersion	Minced muscle	Rat	Fibrosis and scarce skeletal muscle regeneration	[26]
Porcine SIS	Immersion	–	Mouse	Remodeling and partial skeletal muscle regeneration	[27]
Porcine SIS	Immersion	–	Dog	Remodeling and partial skeletal muscle regeneration. No functional recovery	[28]
Porcine SIS; Porcine skeletal muscle	Perfusion	–	Rat	Skeletal muscle regeneration with partial functional recovery. Improved results for skeletal muscle scaffolds	[29]
Porcine SIS; Carbodiimide-crosslinked porcine SIS	Immersion	–	Rat	Skeletal muscle regeneration with partial functional recovery. Improved results for SIS scaffolds	[30]
Porcine SIS; Porcine UBM	Immersion	–	Pig	Remodeling and fibrosis. No functional recovery	[31]
Murine skeletal muscle	Immersion	Co-culture of adult murine or human muscle stem cells and muscle resident cells	Mouse	Functional skeletal muscle regeneration improved after exercise regimen	[32]
Rat skeletal muscle	Patent	–	Rat	Functional skeletal muscle regeneration	[33]
Rat skeletal muscle	Perfusion	–	Mouse	Functional skeletal muscle regeneration	[34]
Rat skeletal muscle	Infusion	Minced muscle vs no cells	Rat	Functional skeletal muscle regeneration improved when cell seeded scaffolds are used	[35]
Porcine UBM	Immersion	–	Mouse	Skeletal muscle regeneration with partial functional recovery	[36]
Rat and primate forearm	Perfusion	Co-culture of C2C12 cells, fibroblasts and HUVEC	Rat	Reperfused vascular tree	[44]

## Abbreviations

3D	tridimensional
ECM	extracellular matrix
FGF	fibroblast growth factor
HGF	hepatocyte growth factor
IGF1	insulin-like growth factor 1
PCL	polycaprolactone
PEG	poly(ethylene glycolic)
PGA	polyglycolic acid
PLGA	poly(lactic-co-glycolic acid)
PLLA	poly(l-lactic acid)
SC	satellite cells
SDS	sodium dodecil sulfate
SIS	Small intestine submucosa matrix
UBM	urinary bladder matrix
VEGF	vascular endothelial growth factor
VML	volumetric muscle loss
